# APOE ε4 Carriage is Associated with Hippocampus-Olfactory Tract Functional Connectivity

**DOI:** 10.21203/rs.3.rs-6753781/v1

**Published:** 2025-06-11

**Authors:** Toshikazu Ikuta, Taylor Bither

**Affiliations:** University of Mississippi; University of Miami

**Keywords:** APOE, Alzheimer’s Disease, Olfaction, Resting State Functional Connectivity

## Abstract

Olfactory dysfunction often emerges before cognitive symptoms and may signal early vulnerability to neurodegenerative processes. This study examined whether genetic risk, specifically the presence of the epsilon 4 allele in apolipoprotein E, is associated with altered functional connectivity between the hippocampus and olfactory-related brain regions. Resting-state functional imaging data from 126 participants (mean age = 71.8 years, SD = 6.9; 67 females) across a range of clinical stages were analyzed. Functional connectivity was computed between the hippocampus and four olfactory-related regions: anterior piriform cortex, posterior piriform cortex, olfactory bulb, and olfactory tract. Multiple regression models assessed whether genetic risk, age, sex, and clinical diagnosis predicted connectivity strength. Genetic risk was significantly associated with increased connectivity between the hippocampus and both the olfactory bulb and olfactory tract, while no significant effects were observed in the piriform cortex regions. Clinical diagnosis was not a significant predictor of connectivity in any region. These results suggest that genetic risk is linked to early functional reorganization in specific olfactory-hippocampal pathways, particularly the olfactory tract, independent of clinical disease stage. The olfactory-hippocampal network may serve as a sensitive target for detecting early brain changes associated with neurodegenerative risk.

## Introduction

The olfactory system is more sensitive to Alzheimer’s Disease (AD) than other parts of the brain. Olfactory dysfunctions are exhibited before cognitive and memory impairments (Wilson et al. 2009). This means that there are parts of the brain that are influenced prior to the onset of the noticeable symptoms. Olfactory deficits have been further found to predict later cognitive decline (Devanand et al. 2015). Smell identification has been suggested to be a useful screening tool for AD (Woodward et al. 2017).

Olfactory deficits correlate with beta-amyloid accumulations. Olfactory dysfunctions have also been found to be associated with beta-amyloid accumulations (Wesson et al. 2010), which has been one of the most studied biomarkers so far. It is plausible that the same underlying pathology responsible for cognitive decline in Alzheimer’s disease also contributes to olfactory deficits.

The hippocampus and olfaction have been long known for their linkage. The hippocampus is considered to be responsible for memory and cognitive decline in AD (DeTure and Dickson 2019; Halliday 2017). Olfaction-Hippocampal functional connectivity was found to be affected in AD (Lu et al. 2019). Olfaction has long been implicated in its close association with the hippocampus (Eichenbaum and Otto 1992). They are anatomically connected. Hippocampal CA1 region has been shown to have olfactory afferents (Biella and de Curtis 2000). Recently, human olfactory loss has been found to reduce hippocampal activation in emotional memory (Han et al. 2019).

Olfactory dysfunction is not unique to AD. Olfactory deficits were found in some other disorders, such as Parkinson’s disease (Doty et al. 1988; Ross et al. 2008), and schizophrenia (Kästner et al. 2013; Moberg et al. 2014); It has been implicated that they are more prevalent for bipolar disorder, depression and autism (Hardy et al. 2012; Kamath et al. 2018; Taalman et al. 2017; Tonacci et al. 2017). Understanding olfactory networks in AD may allow us to deepen our knowledge about broader neurological mechanisms.

Olfaction is also affected by genetic risks. APOE ε4 affects olfaction, and olfaction is more sensitive than memory and cognition. Genetic influence on olfaction has been found in the context of AD. The 4 allele in Apolipoprotein E (APOE) is known as one of the strongest genetic risk (Belloy et al. 2019). Olfactory dysfunction has also been suggested in its association with the APOE. Indeed, association between APOE and olfaction has long been reported (Bacon et al. 1998; Woodward et al. 2017). Olfactory dysfunction is more severe in APOE 4/4 homozygotes than in 3/4 heterozygotes and 3/3 homozygotes (Oleson and Murphy 2015). This suggests that 4 alleles influence olfaction. Heterozygotes of APOE ε4 showed olfactory decline in middle age adults, but did not show cognitive decline (Josefsson et al. 2017). It is implied that olfaction is more sensitive to APOE ε4 than cognition. Knockout of apoE showed olfactory deficiency in rodents (Nathan et al. 2004), further suggesting that the olfactory dysfunction may be a fundamental influence found also in rodents. Understanding olfactory dysfunction may facilitate translating animal models into the human context. Despite clearly known associations between APOE ε4 and olfaction and between APOE ε4 and AD, the underlying mechanism between APOE genotype and AD has not been abundantly understood.

In this study, we aimed to evaluate whether olfaction may contribute to the association between APOE and AD. We tested olfactory functional connectivity as an endophenotype of the APOE-ε4, in order to elucidate the underlying functional network that is influenced by APOE ε4.

## Methods

Data used in this study were obtained from the Alzheimer’s Disease Neuroimaging Initiative (ADNI) database (adni.loni.usc.edu). ADNI was launched in 2003 as a public–private partnership led by Principal Investigator Michael W. Weiner, MD. The original aim of ADNI was to determine whether serial magnetic resonance imaging (MRI), positron emission tomography (PET), other biological markers, and clinical and neuropsychological assessments could be combined to measure the progression of mild cognitive impairment and early Alzheimer’s disease. Current objectives include validating biomarkers for clinical trials, increasing cohort diversity to improve generalizability, and providing open-access data to support research on the diagnosis and progression of Alzheimer’s disease. For the most up-to-date information, please visit adni.loni.usc.edu. Raw MRI files were downloaded as compressed DICOM images and subsequently converted into NIFTI format.

### Preprocessing

Initial data preprocessing followed the procedures outlined in a previous study (Kiparizoska and Ikuta 2017). Data preprocessing and statistical analyses were conducted using the FMRIB Software Library (FSL) and the Analysis of Functional NeuroImages (AFNI). The anatomical volume for each subject was skull-stripped, segmented into gray matter, white matter, and cerebrospinal fluid (CSF), and registered to the MNI152 2mm standard space. Through this registration process, 12 affine transformation parameters were generated to align the resting-state fMRI (rsfMRI) volumes with the MNI152 2mm space, enabling subsequent registration of the processed EPI volumes.

The first four EPI volumes were discarded to allow signal stabilization. Transient signal spikes were removed using de-spiking interpolation. To correct for head motion, each volume was linearly registered to the first remaining volume, from which six motion parameters and the displacement distance between consecutive volumes were estimated. Each rsfMRI volume was then regressed using signals from white matter and CSF, along with the six motion parameters, to minimize physiological and motion-related noise.

Following regression, the data were smoothed using a 6mm full-width at half-maximum (FWHM) Gaussian kernel, resampled, spatially transformed, and aligned to the MNI152 2mm standard brain space. Motion scrubbing was conducted by calculating the root mean square (RMS) deviation of head displacement between successive volumes using a 40mm radius spherical surface, as implemented in FSL’s rmsdiff tool (Power et al. 2015). Volumes exceeding a displacement threshold of 0.3mm were excluded from further statistical analyses (Siegel et al. 2014).

## Registration of the Olfactory Regions

Following the methodology established in a previous schizophrenia study (Kiparizoska and Ikuta 2017), four regions of interest (ROIs)—the olfactory bulb, olfactory tract, anterior piriform cortex, and posterior piriform cortex—were manually segmented in MNI (Montreal Neurological Institute) 2mm space based on anatomical descriptions found in the literature (Gottfried 2010; Howard et al. 2009; Scherfler et al. 2013). These olfactory ROIs were defined within the MNI 2mm standard space.

Although accurate registration of individual functional datasets may result in proper alignment of the olfactory ROIs with their corresponding anatomical regions, all registrations were visually inspected in the MNI-registered anatomical space to ensure precision. Each individual session was evaluated following preprocessing by two authors (TB and TI), who independently inspected all 656 series while remaining blinded to each other’s assessments. The inspection involved verifying the alignment between the MNI-registered anatomical volumes and the MNI 2mm template, as well as confirming the appropriate positioning of the four olfactory ROIs.

Each session was rated on a five-point scale ranging from 1 (significant problems) to 5 (no detectable issues). Upon completion of the visual inspection, evaluations from the two authors were compared by the principal investigator. Any series that received a score of 4 or lower (indicating any level of misalignment or issue) from both raters were excluded from further analysis.

## Connectivity Analysis of the Olfactory Regions

The bilateral hippocampi were defined anatomically using the Harvard-Oxford Subcortical Structural Atlas (Desikan et al., 2006). Resting-state functional connectivity was computed between the hippocampi and each of the four olfactory-related regions: the anterior piriform cortex (APC), posterior piriform cortex (Piri), olfactory bulb (OB), and olfactory tract (OT). ROI-to-ROI connectivity analyses were conducted at the individual subject level, resulting in Fisher’s *Z*-transformed correlation coefficients representing connectivity strength between the hippocampus and each olfactory region. These *Z*-scores were used as dependent variables in linear regression models. Multiple linear regression analyses were conducted to examine whether APOE ε4 allele count, age, sex, and baseline clinical diagnosis predicted hippocampal functional connectivity with four olfactory-related brain regions. These regression models were run separately for each olfactory region.

## Results

A total of 126 participants were included in the analysis. The mean age was 71.8 years (SD = 6.9). The sample included 67 females (53.2%) and 59 males (46.8%). Baseline diagnostic categories were distributed as follows: 26 participants (20.6%) were diagnosed with Alzheimer’s disease (AD), 24 (19.0%) were cognitively normal (CN), 34 (27.0%) had early mild cognitive impairment (EMCI), 23 (18.3%) had late mild cognitive impairment (LMCI), and 19 (15.1%) were classified as having subjective memory complaints (SMC).

For the **anterior piriform cortex**, none of the predictors, including APOE ε4 (*p* = .608) and diagnosis (*p* = .911), were significantly associated with functional connectivity. The overall model was not significant, *F*(4, 120) = 0.09, *p* = .986, and explained minimal variance (*R*^*2*^ = .003).

For the **posterior piriform cortex**, the model remained nonsignificant, *F*(4, 120) = 0.89, *p* = .471, with no significant effects for APOE ε4 (*p* = .203) or diagnosis (*p* = .408). Only the intercept reached significance (*p* = .048), suggesting baseline elevation in connectivity values but no meaningful modulation by predictors.

In contrast, for the **olfactory bulb**, APOE ε4 was significantly associated with increased hippocampal connectivity (*B* = 0.090, *p* = .026), whereas diagnosis again did not contribute significantly (*p* = .988). The overall model approached significance, *F*(4, 92) = 1.47, *p* = .218, with modest explanatory power (*R*^*2*^ = .060).

For the **olfactory tract**, both APOE ε4 (*B* = 0.080, *p* = .022) and age (*B* = 0.010, *p* = .003) were significant predictors of increased connectivity. Diagnosis did not predict connectivity (*p* = .747). The model was statistically significant, *F*(4, 120) = 4.15, *p* = .004, explaining 12.1% of the variance (*R*^*2*^ = .121).

## Discussions

In this study, we investigated whether APOE ε4 carriage is associated with hippocampal functional connectivity to four olfactory-related brain regions—anterior piriform cortex (APC), posterior piriform cortex (PCC), olfactory bulb (OB), and olfactory tract (OT)—after accounting for age, sex, and baseline clinical diagnosis. This finding indicates that individuals with higher APOE ε4 allele count tend to show stronger functional connectivity between the hippocampus and the olfactory tract. In other words, APOE ε4 carriage is associated with increased communication or synchronization between these two brain regions, OT and hippocampus. Additionally, older age was independently associated with greater connectivity in the same pathway. Together, these factors explained about 12% of the variability in connectivity strength, suggesting that both genetic risk and age contribute meaningfully to differences in hippocampal-olfactory tract interactions.

These results indicate that APOE ε4 may be associated with altered hippocampal connectivity in specific olfactory structures, particularly the olfactory tract. The fact that this association remained significant after adjusting for age, sex, and clinical diagnosis suggests that APOE ε4-related changes in functional connectivity may occur independently of observable cognitive status. The olfactory tract, as a major relay structure in the olfactory pathway, may reflect early and genotype-sensitive differences in brain network organization.

Although the association between APOE ε4 and connectivity in the olfactory bulb reached statistical significance, the lack of overall model significance limits the interpretability of this finding. Previous studies have shown mixed results regarding APOE ε4-related changes in olfactory bulb structure and function, with some reporting early degeneration (Wesson et al. 2010) and others noting region-specific variability (Murphy 2019). Further investigation in larger or stratified samples will be necessary to determine whether the olfactory bulb reliably exhibits APOE ε4-related alterations in functional connectivity. The absence of significant findings in the APC and PCC regions aligns with prior work suggesting that piriform cortex involvement may occur later in the disease process (Jones et al. 2011; Sheline and Raichle 2013) or may be less sensitive to early genetic risk factors. This pattern suggests that the effects of APOE ε4 are not uniformly distributed across the olfactory system.

Importantly, we found no significant associations between baseline clinical diagnosis and hippocampal connectivity in any of the four regions examined. This suggests that diagnosis, when modeled as an ordinal continuum from cognitively normal to Alzheimer’s disease, may not correspond linearly with functional alterations in the olfactory-hippocampal network. Prior functional connectivity studies have shown that such changes can precede clinical symptoms and may not correlate directly with diagnostic staging (Jones et al. 2011; Sheline and Raichle 2013). It is possible that these connectivity differences occur independently of clinical stage or that categorical or nonlinear representations of diagnosis are more suitable (Greicius et al. 2004). The lack of a diagnostic effect also supports the use of functional connectivity as a potential early marker of neurodegenerative risk, particularly in genetically at-risk populations (Filippini et al. 2009).

This study has several limitations. First, the cross-sectional design limits our ability to infer the temporal progression of APOE ε4-related connectivity changes or their relationship to future cognitive decline. Longitudinal imaging studies will be necessary to determine whether these patterns predict clinical conversion or progression. Second, while we used an ordinal scale for diagnosis to reflect disease progression, categorical or biomarker-based classifications (e.g., amyloid/tau status) may provide more biologically relevant groupings (Jack Jr. et al. 2018). Finally, our sample size—particularly in stratified diagnostic groups—may have reduced statistical power to detect subtle effects, especially in the piriform cortex, where signal variability is known to be high in functional imaging studies (Kopietz et al. 2009).

## Conclusions

These findings highlight the olfactory tract as a potentially sensitive site for APOE ε4-related functional changes. While the results in the olfactory bulb were strongly suggestive, only the olfactory tract showed a statistically robust association. Further research incorporating longitudinal data and additional biomarkers is needed to determine the temporal and clinical significance of these connectivity patterns.

## Figures and Tables

**Figure 1:**
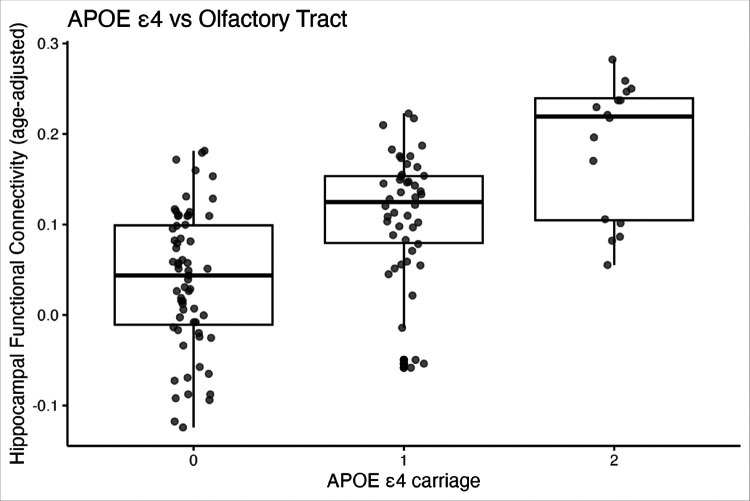
Association between APOE ε4 Carriage and Hippocampus - Olfactory Tract Connectivity

## Data Availability

The original data is available at the NKI-RS website
